# Autoantibodies specific for apoptotic U1-70K are superior serological markers for mixed connective tissue disease

**DOI:** 10.1186/ar1490

**Published:** 2005-01-11

**Authors:** Daniëlle Hof, Kalok Cheung, Dirk-Jan RAM de Rooij, Frank H van den Hoogen, Ger JM Pruijn, Walther J van Venrooij, Jos MH Raats

**Affiliations:** 1 Department of Biochemistry, Nijmegen Centre for Molecular Life Sciences, Radboud University Nijmegen, Nijmegen, The Netherlands; 2 Department of Rheumatology, Sint Maartenskliniek, Nijmegen, The Netherlands; 3 Department of Rheumatology, University Medical Center St Radboud, Nijmegen, The Netherlands; 4 ModiQuest BV, Nijmegen, The Netherlands

**Keywords:** apoptosis, autoantibodies, mixed connective tissue disease, U1 snRNP, U1-70K

## Abstract

Modifications occurring on autoantigens during cell death have been proposed to have a role in the initiation of autoimmune diseases. Patients suffering from mixed connective tissue disease (MCTD) produce autoantibodies directed to U1 small nuclear ribonucleoprotein (snRNP), and antibodies against a 70 kDa protein component, the U1-70K (70K) protein, are the most prominent. During apoptosis, 70K is cleaved by caspase-3 to a 40 kDa product, which remains associated with the complex. Autoantibodies preferentially recognizing the apoptotic form of 70K have been described previously, and an apoptosis-specific epitope on 70K has been identified. This study shows that 29 of 53 (54%) MCTD sera preferentially recognize the apoptotic form of 70K over intact 70K. Moreover, we show that antibodies directed to an apoptosis-specific epitope on 70K are more specifically associated with MCTD than other anti-70K antibodies, suggesting that apoptotic 70K is a better antigen for the detection of these antibodies in MCTD patients. Longitudinal analysis of 12 MCTD patients showed in several patients that early sera are relatively enriched with antibodies recognizing an apoptosis-specific epitope, and that the levels of these apoptosis-specific antibodies decrease in time. These findings indicate that the early detection of apoptotic 70K is of considerable interest for anti-U1 snRNP-positive patients.

## Introduction

Patients suffering from autoimmune diseases are characterized by the presence of autoantibodies directed to a wide range of autoantigens. Mixed connective tissue disease (MCTD) is a relatively rare systemic autoimmune disease and includes a group of patients with overlapping clinical symptoms of systemic lupus erythematosus (SLE), systemic sclerosis (SSc), rheumatoid arthritis and polymyositis/dermatomyositis. Sharp and colleagues were the first to describe MCTD as a distinct rheumatic disease [1], but whether MCTD can be regarded as a distinct disorder has been a subject of discussion [2]. A characteristic serological feature that distinguishes MCTD patients from patients with other connective tissue diseases is high levels of autoantibodies directed against the U1 small nuclear ribonucleoprotein (snRNP) particle [1,3]. The U1 snRNP is a highly conserved RNA–protein complex, located in the nucleus, where it is involved in the processing of pre-mRNA [4,5]. It consists of the U1 snRNA molecule and several proteins: the U1A, U1C and U1-70K (70K) proteins are components specific for the U1 snRNP, whereas the seven Sm proteins (B/B', D1, D2, D3, E, F and G) are shared with other U snRNPs [6]. Most U1 snRNP components are autoantigenic in MCTD and SLE. Autoantibodies directed against U1A, U1C, 70K and the U1 snRNA molecule are mainly found in MCTD patients, whereas autoantibodies targeting Sm-D, Sm-B/B' and the E.F.G complex are more specifically associated with SLE [7,8].

The mechanisms through which such autoantigens, generally highly conserved and ubiquitously expressed molecules, escape tolerance and are recognized by the immune system as non-self remain unclear, but it is proposed that cell death is important in the initiation of autoimmune responses [9,10]. Recently, secondary necrosis has also been put forward as a source of proteolytically modified autoantigens [11], but the modifications that occur on autoantigens during apoptosis were studied most extensively. Apoptotic modifications on autoantigens include specific cleavage by caspases or granzyme B, (hyper)phosphorylation, dephosphorylation, citrullination, methylation and transglutaminase cross-linking [10,12,13], and it is thought that these modifications might be seen by the immune system as novel 'cryptic' epitopes. It is believed that these novel epitopes induce the primary immune response, and that secondary immune responses and epitope spreading result in autoantibodies that are directed against unmodified regions of the autoantigens and antigens that are associated with the initially modified autoantigen [9].

One of the apoptotic modifications occurring on the U1 snRNP is the cleavage of 70K at residue 341 by caspase-3 [14,15]. Antibodies against 70K are in general the first autoantibodies to appear in anti-U1 snRNP (often referred to as anti-RNP) positive patients, indicating that 70K is important as an initial autoantigen [16]. The molecular and immunological characteristics of the major apoptotic isoform of 70K, a 40 kDa cleavage product that remains associated with the U1 snRNP complex [17], and its role in the triggering of the primary and possibly secondary autoimmune response, are therefore intriguing.

Recently it was shown that sera of some anti-U1 snRNP positive patients contain antibodies that specifically bind to the apoptotic form of 70K, which displays an epitope that is not present on the intact form [18,19]. This epitope is dependent on the region between amino acids 180 and 205, partly overlapping with the RNA-binding domain and overlapping with the most common T cell epitope [20]. In this study we analyzed a cohort of MCTD and control patients for the presence of autoantibodies against intact and apoptotic 70K. Moreover, we longitudinally analyzed sera from another group of MCTD patients. Our results show that, early in disease, autoantibodies directed against the apoptotic form of 70K (70K^apop^) are more strongly represented than autoantibodies against the intact form. Longitudinal studies also show that autoantibodies against 70K^apop ^are not significantly correlated with disease flares.

## Methods

### Patient sera

All patients were seen at the Department of Rheumatology of the University Medical Centre Nijmegen or the St Maartenskliniek Nijmegen (The Netherlands), and were classified in accordance with standard criteria for each disease. All MCTD patients (*n *= 53) tested positive for anti-U1 snRNP autoantibodies by counter-immunoelectrophoresis, and for antibodies against one or more components of the U1 snRNP complex by immunoblotting. Most of the sera (91%) were also RNP positive as shown by U1 snRNA co-immunoprecipitation. Longitudinal serum collections were obtained from 12 MCTD patients and have been described previously [21]. From each patient, over a period of 4–15 years (average 10 years), 8 to 33 serum samples (average 18 samples) were available and were analyzed. During the follow-up study, the patients were regularly monitored for clinical and serological parameters. At each visit the disease activity was measured in accordance with a validated SLE disease activity index described by Ter Borg and colleagues [22]. Medication was given as indicated by the clinical status. Additionally, patient sera were collected from SLE (*n *= 48), polymyositis/dermatomyositis (*n *= 26), primary Sjögren's syndrome (*n *= 18), SSc (*n *= 10), rheumatoid arthritis (*n *= 3), Raynaud's phenomenon (*n *= 3) and undefined connective tissue disease (*n *= 1). Informed consent was obtained from all participants in accordance with the medical ethical regulations of the local ethics committee. Sera were stored at -70°C until use.

### Cell lines, induction of cell death and preparation of cell extracts

Jurkat (human T cell leukemia) suspension cells were grown in RPMI 1640 medium (Gibco-BRL), supplemented with 1 mM sodium pyruvate, 1 mM penicillin, 1 mM streptomycin and 10% heat-inactivated fetal calf serum (Gibco-BRL), in a humidified 37°C incubator containing 5% CO_2_. Cells were maintained at a concentration of 10^6 ^cells/ml and were induced to undergo apoptosis by the addition of 10 μg/ml anisomycin. Eight hours after induction, apoptotic cells were harvested by centrifugation at 800 *g *for 10 min and washed with PBS. Apoptotic and non-apoptotic Jurkat cells were resuspended in Nonidet P40 (NP40)-containing lysis buffer (25 mM Tris-HCl, pH 7.6, 100 mM KCl, 10 mM MgCl_2_, 0.25 mM dithioerythritol, 1% NP40, Complete™ protease inhibitor cocktail [Roche]) at a concentration of 10^8 ^cells/ml. Cells were lysed on ice for 30 min and subsequently centrifuged for 30 min at 12,000 *g *and 4°C. Supernatants were used immediately or stored at -70°C.

### SDS–polyacrylamide gel electrophoresis and western blotting

Cell extracts of 1.3 × 10^7 ^non-apoptotic Jurkat cells and 1.3 × 10^7 ^apoptotic Jurkat cells, either separately or mixed, were separated by SDS–polyacrylamide gel electrophoresis. Directly after gel electrophoresis, proteins were transferred to a nitrocellulose membrane (Schleicher & Schuell) by semi-dry electroblotting. Staining of the membrane with Ponceau S (Sigma) was used to verify protein transfer.

### Probing western blots with patient sera

All incubation steps were performed at approximately 20°C on a shaking table. Western blots containing non-apoptotic and apoptotic Jurkat cell extracts were pre-blocked with 5% non-fat dried milk in PBS containing 0.1% NP40 (MPBS/NP40) for 2 hours. Subsequently, membranes were incubated with patient serum, diluted 1000–5000-fold in MPBS/NP40, for 1 hour. After extensive washing with PBS containing 0.1% NP40 (PBS/NP40), membranes were incubated with horseradish peroxidase-labeled rabbit anti-human IgA/IgG/IgM antibody (Dako Immunoglobulins), diluted 1000-fold in MPBS/NP40, for 1 hour. After several washes with PBS/NP40 and PBS, bound antibodies were detected by enhanced chemiluminescence. Antibody reactivities against 70K and 70K^apop ^were scored ranging from 0 to 5 by three researchers independently. In each experiment several control antibodies were used.

## Results

**Figure 1:**
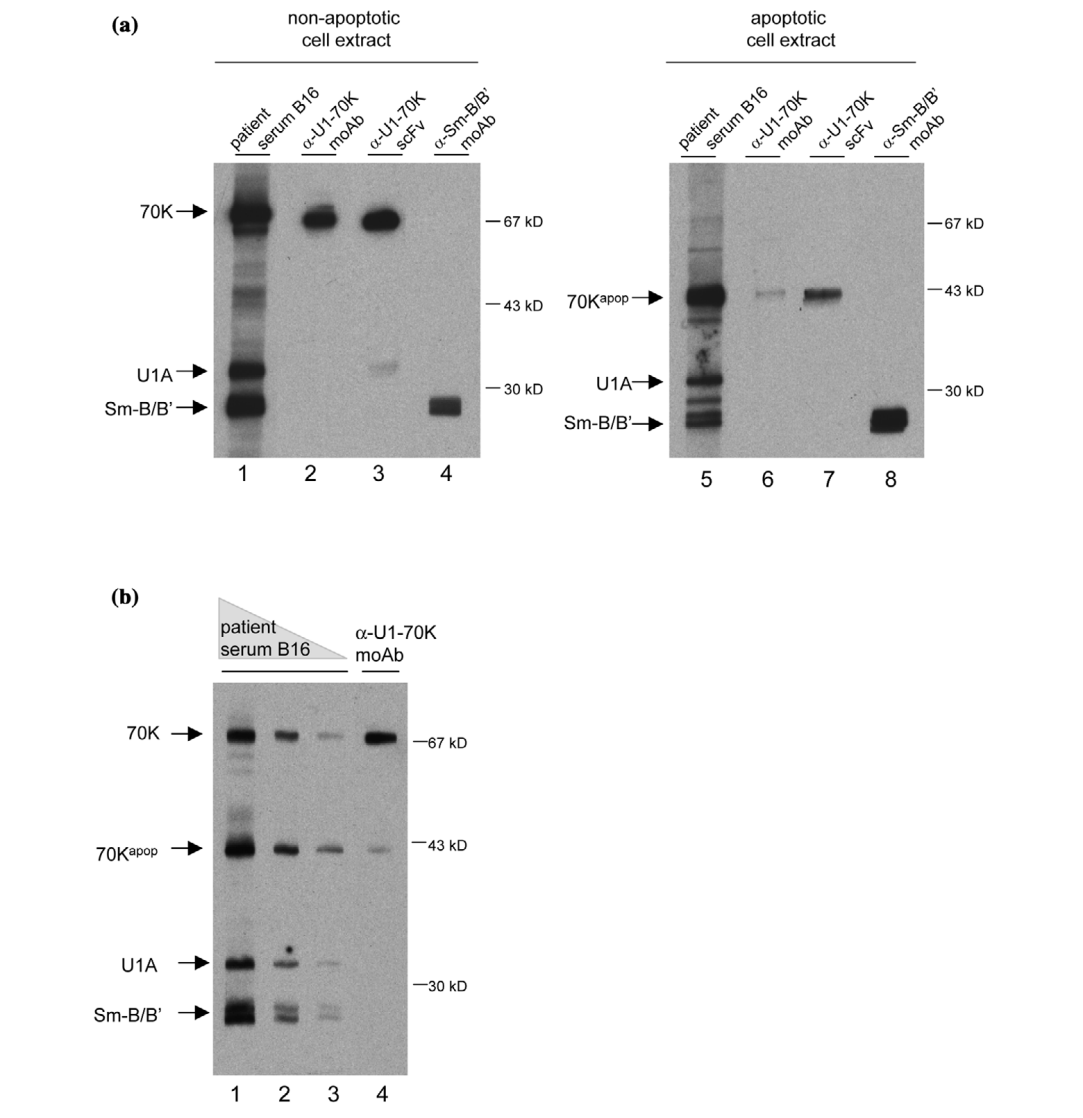
Anti-U1-70K and anti-70K^apop ^detection by western blotting. Apoptosis was induced in Jurkat cells by incubation with anisomycin for 8 hours. Western blots were prepared with the resulting cell extracts, and the positions of relevant polypeptides were revealed with patient sera and monoclonal antibodies with the use of a chemiluminescent detection procedure. The positions of the various proteins are indicated on the left, and molecular mass marker positions on the right. **(a) **U1-70K (70K) detected with a serum from MCTD patient B16 (lanes 1 and 5), anti-70K monoclonal antibody 2.73 (lanes 2 and 6) and an anti-70K single-chain recombinant antibody (scFv; lanes 3 and 7) (70K); lanes 4 and 8, Sm-B/B' detected with a monoclonal anti-Sm-B/B' antibody (ANA125); the position of U1A, which is also recognized by serum from MCTD patient B16 (lanes 1 and 5), was determined by a U1A-specific monoclonal antibody (not shown). In apoptotic cells (lanes 5–8), 70K is present as a 40 kDa species (70K^apop^). **(b) **A serum sample from MCTD patient B16 was applied at 5000-fold (lane 1), 10,000-fold (lane 2) and 20,000-fold (lane 3) dilution on a western blot containing a mixture of non-apoptotic and apoptotic Jurkat cell extracts. In lane 4 the 70K protein was detected with mouse monoclonal antibody 2.73, which reacts much more efficiently with 70K than with 70K^apop^.

In this study, patient sera were analyzed for the presence of autoantibodies against 70K and its apoptotic product (70K^apop^), on western blots containing extracts of non-apoptotic and apoptotic Jurkat cells. Two positive controls for the detection of 70K and 70K^apop ^were included in each experiment: anti-70K mouse monoclonal antibody 2.73 [23], which displays higher reactivities with 70K than with 70K^apop^, and serum from MCTD patient B16, which reacts with both 70K and 70K^apop^. The position of 70K^apop ^on western blots was confirmed by a recombinant monoclonal antibody recognizing both 70K and 70K^apop ^(Fig. 1a) [24]. The results show that in these apoptotic cells 70K is converted almost completely into 70K^apop^. Besides positive controls for 70K and 70K^apop^, mouse monoclonal antibody ANA125 directed against Sm-B/B' (Fig 1a), and anti-U1-A/U2-B" mouse monoclonal antibody 9A9 (not shown) were also used. To be able to detect autoantibody reactivities to the intact 70K and its apoptotic 40K fragment simultaneously and to facilitate a direct comparison of these reactivities, a mixture of apoptotic and non-apoptotic cell extracts was used to prepare western blots. An additional advantage of this approach was that differences between blots could be excluded, thereby allowing a more accurate comparison of reactivities with 70K and 70K^apop ^in a single patient serum. Serum antibody reactivities against 70K and 70K^apop ^were scored ranging from 0 to 5. Figure 1b shows a western blot containing such a mixture of non-apoptotic and apoptotic Jurkat cell extracts, probed with a serial dilution of serum from MCTD patient B16. It can be seen that the signals for 70K and 70K^apop ^increase when the serum is applied at a lower dilution, indicating that the western blot assay can be used for semi-quantitative interpretation.

### Autoantibodies against 70K are more easily detected with 70K^apop^

The presence of high levels of autoantibodies directed against components of the U1 snRNP, such as 70K, is one of the criteria for the diagnosis of MCTD [2]. However, anti-70K antibodies are also found in some SLE and SSc patients [3]. To compare the disease specificity of anti-70K^apop ^and anti-70K autoantibodies, sera from a group of MCTD patients and from a group of patients suffering from a variety of autoimmune disorders were analyzed. As shown in Table 1, most MCTD patients (54%) displayed antibody reactivities that preferentially recognized 70K^apop ^over the intact 70K protein. Seven patients (13%) reacted with 70K and 70K^apop ^with similar efficiencies, and only 6% of the MCTD patients reacted preferentially with the intact 70K protein. Fourteen sera (27%) did not react detectably with either 70K polypeptide, although the sera were anti-RNP positive by several techniques. These results indicate that 70K^apop ^is a better antigen than the intact 70K protein for the detection of anti-70K autoantibodies. Antibody reactivity with 70K^apop ^was found in only 2% of sera from control groups, whereas antibody reactivity with 70K was found in 5% of patient sera from control groups.

### Autoantibodies against 70K^apop ^are not correlated with disease activity

It has been described that, in some patients with MCTD, antibody titers against the U1 snRNA molecule are correlated with disease activity, and could even possess prognostic value [21]. In contrast, most studies did not find a correlation between disease activity and antibody responses to 70K, either by serum analysis using recombinant protein as antigen in ELISA [21,25] or by analysis on western blots using native protein from cell extracts [26]. Only one study, using ELISA with recombinant 70K as technique, has reported decreasing disease activity concomitant with decreasing anti-70K antibody levels [27]. Because apoptotic modifications on autoantigens, such as the cleavage of 70K, are believed to be involved in the primary autoimmune response, we proposed that immune complexes containing anti-70K^apop ^antibodies might also be important for triggering disease flares. Serum samples were collected longitudinally from 12 MCTD patients by a follow-up during variable time intervals (4–15 years; average 10 years). All samples were analyzed for the presence of autoantibodies against 70K and 70K^apop ^on western blots containing non-apoptotic and apoptotic Jurkat cell extracts, and the presence of these autoantibodies was compared with the disease activity of each patient. The overall conclusion of this longitudinal study was that no significant correlations between antibody titres against either 70K^apop ^or 70K and disease exacerbations could be observed.

### Autoantibodies against 70K^apop ^are more prevalent early in disease

**Figure 2:**
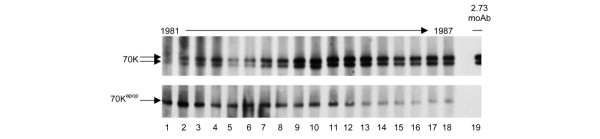
Longitudinal anti-70K analysis of patient T2. Eighteen serum samples taken over a period of 7 years with approximately equal time intervals were analyzed on western blots containing non-apoptotic and apoptotic Jurkat cell extracts. The positions of U1-70K (70K), of which two isoforms are visible, and 70K^apop ^are indicated on the left. In lane 19, 70K was detected with mouse monoclonal 2.73, which reacts much more strongly with 70K than with 70K^apop^. MoAb, monoclonal antibody.

As mentioned above, it has been proposed that apoptotic modifications trigger the primary immune response towards self proteins and that, through secondary immune responses and epitope spreading, autoantibodies directed against unmodified regions on the autoantigen appear at later stages of the disease. To investigate this possibility for the 70K autoantigen, the longitudinal serum collection [21] of 12 MCTD patients was re-examined, now for antibodies against 70K and for antibodies against 70K^apop^. Three patients produced antibodies reacting strongly with 70K^apop^, whereas no or only weak reactivity against 70K was observed. In one of these three patients, autoantibodies against 70K^apop ^were more prevalent in early serum samples, and the level decreased in time. Eight patients were found to have high titres of antibodies with reactivities to both 70K and 70K^apop^. Interestingly, in three of these patients early serum samples showed a higher reactivity with 70K^apop ^than with 70K, whereas later samples showed comparable reactivities with both antigens, or higher reactivities with 70K. An example of this type of reactivity profile is shown in Fig. 2. One of the 12 patient sera did not detectably contain antibodies directed against 70K or 70K^apop^. These results thus support the idea that antibodies against 70K^apop ^appear earlier in the disease than antibodies against the complete 70K protein.

## Discussion

Greidinger and colleagues recently showed that antibodies against the 70K protein in RNP-positive patients are often accompanied by antibodies directed against the apoptotic cleavage product of this autoantigen and that the B cell epitopes recognized on the apoptotic product are antigenically different from those contained in the intact form of the 70K protein [18,19]. This study is the first to confirm and extend these findings and strongly suggests that the reactivity of a patient serum with anti-70K antibodies depends on the presence of antibodies against epitopes shared by 70K and 70K^apop^, and on the presence of antibodies against epitopes exclusively present on 70K^apop^. The major apoptosis-specific epitope on 70K has been shown to be located in the region containing the RNA-binding domain, and its formation depends on amino acids 180–205, overlapping with the most common T cell epitope [20]. Other apoptosis-specific epitopes on 70K have not yet been described. In our recent studies, monoclonal recombinant human antibodies against 70K were isolated from phage display libraries derived from SLE patients, and several of these monoclonal antibodies preferentially recognized 70K^apop ^on a western blot and in immunoprecipitation experiments ([24], and D Hof, unpublished results). It is believed that the apoptotic cleavage of 70K leads to the exposure of a neo-epitope that, if presented to the immune system, triggers the autoimmune response. Greidinger and colleagues showed that a mutant consisting of the amino-terminal 205 amino acids, was indeed able to induce an anti-70K^apop ^antibody response in mice, with subsequent epitope spreading. Interestingly, some of the immunized mice developed pulmonary lesions comparable to lesions found in lungs of MCTD patients. This finding supports the hypothesis that apoptosis-specific epitopes, and antibodies directed against them, might have a pathological role in the triggering and maintenance of the human autoimmune response to 70K [19].

In our study, a minority of MCTD sera (4%) contained autoantibodies exclusively reacting with intact 70K. We suggest that these sera derive from patients in a relatively late disease phase and primarily contain antibodies resulting from expanded epitope spreading. Most epitopes recognized by these sera might therefore be dependent on the carboxy-terminal part of the protein, which is cleaved off during apoptosis and is not present on 70K^apop^. Patients that tested negative in our western blot experiments might either have low levels of anti-70K antibodies or might not produce such antibodies at all. Instead, other components of the U1 snRNP, such as the U1 RNA molecule, U1A or U1C, might be targeted by these sera and might explain their anti-U1 snRNP reactivity.

We show here that most U1 snRNP-positive patient sera preferentially recognize 70K^apop^, which is most probably explained by the presence of antibodies targeting an apoptotic 70K epitope. These results are in line with reports by Greidinger and colleagues [18,19], who found that about 50% of their RNP-positive sera contained 70K^apop ^autoantibodies.

How disease flares are induced is not completely understood. Correlations between serum levels of certain autoantibodies and disease activity have been reported for MCTD and SLE [21,22], but it can be disputed whether these antibodies contribute to the disease flares or are merely epiphenomena. Our data show that antibodies against 70K^apop ^are not significantly correlated with disease activity, suggesting that there is no important role for 70K^apop ^in the initiation of disease flares. However, it is possible that the variations in antibody levels against the apoptosis-specific epitope are masked by the presence of antibodies against other epitopes on 70K and U1-70K^apop^. Furthermore, a polyspecific secondary antibody was used to detect bound serum antibodies, and as a consequence variations in isotype-specific antibody levels might have remained undetected.

It has been reported that the first autoantibodies to appear in anti-RNP-positive patients are generally antibodies against 70K [16,27]. Our results suggest that 70K^apop ^drives the primary autoimmune response to 70K, because in several patients antibodies against an epitope associated with 70K^apop ^preceded the appearance of reactivity with intact 70K. The fact that the first serum samples from relatively few patients exclusively contain anti-70K^apop ^antibodies might be due to the stage of disease development at which the patient enters the rheumatological clinic. It is likely that the first symptoms, later followed by the diagnosis of the disease, had been established years before the start of the longitudinal study. Moreover, it is possible that autoantibodies, especially those generated by the primary immune response, were already present before the manifestation of clinical symptoms and that subsequent epitope spreading might have occurred before the patient entered the rheumatological clinic. For example, anti-cyclic citrullinated peptide autoantibody is a very specific marker for rheumatoid arthritis, and such antibodies can be detected in patients up to 10 years before the occurrence of the first clinical symptoms [28,29]. In our opinion this might explain why a relative enrichment of anti-70K^apop ^antibodies could not be detected in the early sera of all patients.

During apoptosis, the U1 snRNP complex is modified in several ways. In addition to cleavage of 70K, U1 snRNA and the Sm-F protein are cleaved, and phosphorylated serine–arginine proteins associate with the complex [30]. Apoptotic modifications of the U1A and U1C proteins have not yet been described. 70K can be cleaved by caspase-3 and granzyme B, and it can be oxidatively fragmented in the presence of metals, resulting in products of 40, 60 and 55 kDa, respectively. Correlations between the recognition of specific 70K fragments and disease manifestations are interesting. For example, patients suffering from Raynaud's phenomenon preferentially recognize the oxidatively modified 55 kDa fragment of 70K [31]. The findings that early MCTD sera are enriched for antibodies against the 40 kDa apoptotic fragment (70K^apop^) and that most sera show a higher reactivity with this fragment suggest that caspase-3 cleaved 70K has a role in breaking tolerance in these patients. Although granzyme B is postulated to have a role in breaking tolerance [32] to 70K, it is unknown whether specific patient groups preferentially recognize the 60 kDa cleavage product generated by granzyme B, which would be interesting to study in more detail.

## Conclusions

Analysis of a group of MCTD patient sera by western blotting showed that the majority of patient sera recognized 70K^apop ^more efficiently than the intact form of the 70K protein. The fact that the presence of these antibodies in most patients precedes the occurrence of other anti-70K antibodies suggests that 70K^apop ^is particularly important for the early detection of this disease in patients.

## Abbreviations

70K = U1-70K; 70K^apop ^= apoptotic cleavage product of U1-70K protein of about 40 kDa; MCTD = mixed connective tissue disease; NP40 = Nonidet P40; RNP = ribonucleoprotein; SLE = systemic lupus erythematosus; snRNP = small nuclear ribonucleoprotein; SSc = systemic sclerosis; U1-70K = 70 kDa protein component of the U1 snRNP complex.

## Competing interests

JMHR works and holds shares in ModiQuest BV, a company producing antibodies for research purposes, but will not gain or lose financially from publication of this paper.

## Authors' contributions

DH conceived of the study, participated in the design of the study and was involved in the analysis of the immunoassay results. KC performed and analyzed the immunoassays. DR collected the patient sera. FH provided patient data. GP participated in the design of the study and in the analysis of the immunoassay results. WV conceived of the study and participated in the design of the study. JR conceived of the study and was involved in its design and coordination. All authors read and approved the final manuscript.

## Figures and Tables

**Table 1:** Recognition of U1-70K (70K) and its apoptotic cleavage product (70K^apop^) by patients with different autoimmune disorders

		No. (%) of patients with disorder				
		
Patient group (disease)	No. of patients	70K^apop^	70K^apop ^> 70K	70K^apop ^= 70K	70K > 70K^apop^	70K
MCTD	53	15 (28)	14 (26)	7 (13)	1 (2)	2 (4)
SLE	48	0	1 (2)	1 (2)	0	1 (2)
Non-SLE^a^	61	0	0	0	0	2 (3)

^a^The control group of non-systemic lupus erythematosus (non-SLE) patients consists of patients suffering from polymyositis/dermatomyositis (*n *= 26), primary Sjögren's syndrome (*n *= 18), systemic sclerosis (*n *= 10), rheumatoid arthritis (*n *= 3), Raynaud's phenomenon (*n *= 3) and undefined connective tissue disease (*n *= 1). MCTD, mixed connective tissue disease.
